# Evidence of abnormality in glutathione metabolism in the airways of preterm born children with a history of bronchopulmonary dysplasia

**DOI:** 10.1038/s41598-023-46499-w

**Published:** 2023-11-09

**Authors:** Christopher W. Course, Philip A. Lewis, Sarah J. Kotecha, Michael Cousins, Kylie Hart, Kate J. Heesom, W. John Watkins, Sailesh Kotecha

**Affiliations:** 1 Department of Child Health, School of Medicine, Cardiff University, Heath Park, Cardiff, CF14 4XN UK; 2 Faculty of Life Sciences, University of Bristol, Bristol, UK; 3 Department of Paediatrics, Cardiff and Vale University Health Board, Cardiff, UK

**Keywords:** Metabolic pathways, Metabolomics, Respiratory tract diseases, Paediatric research

## Abstract

Preterm-born children are at risk of long-term pulmonary deficits, including those who developed bronchopulmonary dysplasia (BPD) in infancy, however the underlying mechanisms remain poorly understood. We characterised the exhaled breath condensate (EBC) metabolome from preterm-born children, both with and without BPD. Following spirometry, EBC from children aged 7–12 years, from the Respiratory Health Outcomes in Neonates study, were analysed using Time-of-Flight Mass Spectrometry. Metabolite Set Enrichment Analysis (MSEA) linked significantly altered metabolites to biological processes. Linear regression models examined relationships between metabolites of interest and participant demographics. EBC was analysed from 214 children, 144 were born preterm, including 34 with BPD. 235 metabolites were detected, with 38 above the detection limit in every sample. Alanine and pyroglutamic acid were significantly reduced in the BPD group when compared to preterm controls. MSEA demonstrated a reduction in glutathione metabolism. Reduced quantities of alanine, ornithine and urea in the BPD group were linked with alteration of the urea cycle. Linear regression revealed significant associations with BPD when other characteristics were considered, but not with current lung function parameters. In this exploratory study of the airway metabolome, preterm-born children with a history of BPD had changes consistent with reduced antioxidant mechanisms suggesting oxidative stress.

## Introduction

Bronchopulmonary dysplasia (BPD), also known as chronic lung disease of prematurity, is the one of the commonest respiratory consequences of preterm birth and, despite advances in neonatal care over the last twenty years, rates of BPD are continuing to rise^1^. The pathogenesis of BPD is multifactorial, but pulmonary injury secondary to oxygen free radical production and inflammation form an important common pathway leading to altered lung development^2, 3^. A history of preterm birth, both with and without a history of BPD, has been consistently associated with poorer lung function in later life^4^, and there is growing evidence that those with a history of BPD risk the development of chronic obstructive pulmonary disease (COPD) in early adulthood^5, 6^, as well as being diagnosed with asthma^7^, although there is increasing recognition that prematurity-associated lung disease (PLD) has different underlying mechanism to asthma^8^. Lung function continues to develop throughout childhood and adolescence, with increasing number of alveoli, airway size and lung volume^9^, before declining after early adulthood^10^. Preterm-born individuals have been shown to have significantly lower forced expiratory volume in 1 s (FEV_1_) than those born at term^11^, therefore providing a potential therapeutic window of opportunity for optimizing peak lung function, and highlights the importance of understanding the underlying mechanisms of PLD during childhood.

Exhaled breath condensate (EBC) is a useful sample type to study in children as it is easily and non-invasively collected. EBC is composed of droplets of epithelial lining fluid (ELF), evolved from all compartments of the lung during tidal breathing. ELF is a complex matrix of compounds, which includes metabolites, and reflects lung tissue biology^12^. Metabolomic methods simultaneously analyse the entire low-molecular weight (< 2000 Da) metabolite content of biological samples and have been applied extensively to EBC in the study of both adult and paediatric respiratory diseases such as COPD, asthma and cystic fibrosis (CF)^13^, showing the ability to discriminate between asthma phenotypes, and evidence of medication altering the EBC metabolome in CF. It is clear metabolomics offers a tool to unravel mechanisms of disease pathogenesis and progression, and identify potential groups of biomarkers in respiratory pathologies. Metabolomic techniques have previously been used on tracheal aspirates obtained during the neonatal period to study the pathogenesis of BPD^14^, finding an increase in metabolites related to hypoxic stress and nitric oxide synthesis.

We hypothesized that the EBC of preterm-born school-aged children with a history of BPD would show altered metabolite content when compared to preterm-born and term-born control children. Therefore, in this exploratory study, we aimed to characterise the metabolome of preterm-born school-aged children with a history of BPD compared to preterm-born and term-born controls.

## Methods

### Participants

This study was conducted on a cohort of children recruited to the Respiratory Health Outcomes in Neonates study (RHiNO, EudraCT: 2015-003712-20) which has been described extensively previously^15–17^. In brief, children from a previous study^18^ were supplemented with additional preterm-born children identified by the NHS Wales Informatics Service and sent a respiratory and neurodevelopmental questionnaire if they were born ≤ 34 or ≥ 37 weeks’ gestation and were aged 7–12 years. Children with significant congenital malformations, cardiopulmonary or neuromuscular disease were excluded. Ethical approval was obtained from the South-West Bristol Research Ethics Committee (15/SW/0289). Parents gave informed written consent and children provided assent. The study was conducted according to the Good Clinical Practice (GCP) guidelines and the Declaration of Helsinki.

Following a home assessment, a subset of responders attended the hospital-based children’s research facility for comprehensive clinical examination and respiratory testing including collection of EBC, conducted by a trained nurse and paediatrician between January 2017 and November 2019. Spirometry (MasterScreen Body and PFT systems, Vyaire Medical, Germany) was performed to ATS/ERS guidelines^19^ and normalised using Global Lung Initiative (GLI) references^20^. Any respiratory medications were withheld prior to their assessment (short- and long-acting β2-agonists for 8- and 48-h respectively; inhaled corticosteroids for 24 h; and leukotriene receptor antagonists for 48 h) and children were free of respiratory infections for at least three weeks prior to testing. Term-born children who had %FEV_1_ > 90% were included as term controls. BPD was defined as oxygen-dependency of 28 days or greater for those born < 32 weeks’ gestation and at 56 days of age for those born ≥ 32 weeks’ gestation)^21^. Intrauterine growth restriction (IUGR) was defined as birthweight < 10th percentile adjusted for sex and gestation (LMS Growth version 2.77, Medical Research Council, UK). Doctor-diagnosed asthma was self-reported by parents. Neonatal history was corroborated with medical records. Socioeconomic status was assessed using the Welsh Index of Multiple Deprivation^22^ scores from 2019, the most contemporaneous available for this cohort.

### EBC sampling

EBC was collected in a standardised manner using a cooling tube (RTube®, Respiratory Research Inc., Texas, USA), that was pre-cooled to − 20 °C for at least two hours prior to use, during 10 min of passive tidal breathing, with the participant wearing a nose clip, stopping briefly to swallow saliva if needed, as per manufacturer’s instructions. Environmental temperature and humidity remained stable during sampling. The RTube® is a single-patient, single-use design, preventing cross-contamination, and features a large ‘Tee’ section to separate saliva from exhaled breath, thereby ensuring collection of ELF and not oropharyngeal secretions. EBC was collected immediately prior to spirometry and, once collected, samples were immediately separated into aliquots and stored at − 80 °C pending analysis.

### Metabolome analysis

EBC samples were analysed using Gas Chromatography Time-of-Flight Mass Spectrometry (GCTOF-MS) by the West Coast Metabolomics Centre (University of California, Davis), who have previously published their analytical method^23^. 50 μL of each sample was fractionated using an Agilent 6890 gas chromatograph (Agilent, Santa Clara, CA, USA), controlled using Leco ChromaTOF software v2.32 (LECO, St. Joseph, MI, USA), in a Rtx-5Sil MS (Restek, Bellafonte, PA, USA) column (30 m length ×  0.25 mm internal diameter with 0.25 μm film made of 95% dimethyl/5%diphenylpolysiloxane). Column temperature was maintained between 50 and 330 °C, with a helium mobile phase. Injection volumes of 0.5 μL were used, with injection temperatures starting at 50 °C, ramped up to a maximum temperature of 250 °C by 12 °C s^−1^. Oven temperature program was set to 50 °C for 1 min, then ramped at 20 °C min^−1^ to 330 °C, and held constant for 5 min. The analytical GC column was protected by a 10 m long empty guard column which was cut by 20 cm intervals whenever the reference mixture QC samples indicated problems caused by column contamination. This sequence of column cuts has previously been validated, with no detrimental effects being detected with respect to peak shapes, absolute or relative metabolite retention times or reproducibility of quantifications. This chromatography method yields excellent retention and separation of primary metabolite classes (amino acids, hydroxyl acids, carbohydrates, sugar acids, sterols, aromatics, nucleosides, amines and miscellaneous compounds) with narrow peak widths of 2–3 s and very good within-series retention time reproducibility of better than 0.2 s absolute deviation of retention times. Automatic liner exchanges after each set of 10 injections were used, which reduces sample carryover for highly lipophilic compounds.

All spectra were acquired using a Leco Pegasus IV (LECO, St. Joseph, MI, USA) time of flight mass spectrometer, with unit mass resolution at 17 spectra s^−1^ from 80 to 500 Da at − 70 eV ionization energy and 1800 V detector voltage with a 230 °C transfer line and a 250 °C ion source. Raw data files were normalised to QC/pool samples using the systematic error removal by random forest (SERRF) method^24^. Raw data files were processed and metabolites identified with the BinBase metabolomics database^25^, using an algorithm based on the following: validity of chromatogram (< 10 peaks with intensity > 10^7^ counts s^−1^), unbiased retention index marker detection (MS similarity > 800, validity of intensity range for high m/z marker ions), retention index calculation by 5th order polynomial regression. Spectra were cut to 5% base peak abundance and matched to database entries from most to least abundant spectra using the following matching filters: retention index window ± 2000 units (equivalent to about ± 2 s retention time), validation of unique ions and apex masses (unique ion must be included in apexing masses and present at > 3% of base peak abundance), mass spectrum similarity fitted criteria dependent on peak purity and signal/noise ratios and a final isomer filter. Quantification of metabolites were reported as spectral peak height of the unique ion detected (m/z value) at the specific retention index. Peak heights are more precise for low abundant metabolites than peak areas, due to the larger influence of baseline determinations on areas compared to peak heights.

### Statistical analysis

Sample demographics were compared using chi-squared or one-way ANOVA with Bonferroni correction tests as appropriate. Metabolite quantities were log_10_ transformed and visually inspected for normality. Metabolites with mean and median peak intensities below the mass spectrometer’s limit of detection were removed from further analysis to ensure robust statistical comparisons between clinical groups. Fold changes between groups were calculated and log_2_ transformed (log_2_FC) for visualization. Independent t-test/ANOVA with post-hoc Bonferroni correction was used to compare metabolite quantities between groups. Metabolite Set Enrichment Analysis (MSEA; identifying biological processes linked to over-represented metabolites) was performed on all metabolites identified with a significantly different quantity between groups using the Small Molecule Pathways Database (SMPDB)^26^, which is based on the Human Metabolome Database (HMDB). Univariable and multivariable linear regression models were used to identify associations between participant characteristics and metabolites of interest identified by MSEA. p < 0.05 was considered statistically significant. All analyses were performed using R v4.0.4 (R Foundation for Statistical Computing, Austria) and MetaboAnalyst v5.0 (www.metaboanalyst.ca)^27^.

## Results

From 1426 returned questionnaires and 768 who underwent home assessments, a total of 241 children underwent detailed assessment at the research facility. EBC was successfully collected and analysed from 214 (89%) children with adequate spirometry (Supplementary Fig. 1). Sample demographics are shown in Table 1. 34 preterm-born children had a diagnosis of BPD (13 mild BPD, 21 moderate/severe BPD)^21^. Preterm-born children with a history of BPD (BPD) were born at a significantly lower gestational age compared to those without BPD (No BPD) (mean ± SD 27.1 ± 2.1 weeks vs 31.8 ± 1.9, p =  < 0.001), with a significantly lower birthweight (1029 ± 415 g vs 1817 ± 493, p =  < 0.001). No BPD group was significantly older than the Term group at assessment (10.3 ± 1.1 years vs 9.7 ± 1.1, p = 0.002) but there was no significant age difference between the BPD and the No BPD and Term groups. There was no difference in socioeconomic deprivation scores between the three groups. Percent predicted forced expiratory volume in 1 s (%FEV_1_) was significantly lower in the BPD group compared to both the No BPD (86.9 ± 15.9 vs 93.2 ± 14.1, p = 0.036) and Term groups (86.9 ± 15.9 vs 104.3 ± 7.1, p =  < 0.001). FEV_1_/Forced Vital Capacity (FVC) ratio was also significantly lower in the BPD group compared to the No BPD (0.77 ± 0.10 vs 0.82 ± 0.09, p = 0.002) and Term groups (0.77 ± 0.10 vs 0.85 ± 0.06, p =  < 0.001). Percent predicted mid-expiratory flows (%FEF_25-75_) were also significantly lower in the BPD group compared to No BPD (64.0 ± 25.8 vs 79.3 ± 24.6, p = 0.003) and Term groups (64.0 ± 25.8 vs 94.7 ± 19.1, p =  < 0.001).

**Table 1:** Participant demographics.

Variable	Preterm BPD n = 34	Preterm No BPD n = 110	Term n = 70
Sex (male), n (%)	15 (44.1)	54 (49.1)	37 (52.9)
Ethnicity (white), n (%)	32 (94.1)	103 (93.6)	69 (98.6)
Gestational age (weeks), mean (SD)	27.1 (2.1)***^†††^	31.8 (1.9)^†††^	40.0 (1.1)
Birthweight (g), mean (SD)	1029 (415)***^†††^	1817 (493)^†††^	3528 (518)
Birthweight (z-score), mean (SD)	-0.06 (1.29)	0.17 (1.38)	0.08 (0.97)
Antenatal Steroids, n (%)	28 (84.8)^†††§^	94 (88.7)^†††§^	0 (0)
Intrauterine growth restriction, n (%)	8 (23.5)^††^	18 (16.4)^†^	4 (5.7)
Age at testing (years), mean (SD)	9.9 (1.4)	10.3 (1.1)^††^	9.7 (1.1)
Weight (kg), mean (SD)	36.3 (13.1)	37.7 (9.0)	36.6 (10.5)
Weight (z-score), mean (SD)	0.08 (1.54)	0.31 (1.02)	0.46 (1.02)
Body Mass Index (kg/m^2^), mean (SD)	18.1 (4.1)	18.0 (3.1)	17.9 (3.2)
Body Mass Index (z-score), mean (SD)	0.14 (1.52)	0.14 (1.24)	0.30 (1.08)
Asthma diagnosis, n (%)	9 (36.0)	22 (20.0)	5 (7.1)**
WIMD 2019 Rank, mean (SD)	1019 (507)	1052 (545)	1178 (520)
FEV_1_ (%predicted), mean (SD)	86.9 (15.9)*^†††^	93.2 (14.1)^†††^	104.3 (7.1)
FVC (%predicted), mean (SD)	99.2 (10.7)^††^	99.1 (11.6)^†††^	107.6 (8.8)
FEV_1_/FVC, mean (SD)	0.77 (0.10)**^†††^	0.82 (0.09)	0.85 (0.06)
FEF_25-75%_ (%predicted), mean (SD)	64.0 (25.8)**^†††^	79.3 (24.6)^†††^	94.7 (19.1)
Total volume of EBC collected (ml), mean (SD)	1.1 (0.3)^†^	1.1 (0.4)	1.3 (0.3)

Comparisons by ANOVA with Bonferroni correction/Chi-squared test as appropriate at baseline.

WIMD, Welsh Index of Multiple Deprivation 2019 Rank Scores.

BPD, Bronchopulmonary Dysplasia.

FEV_1_, Forced Expiratory Volume in 1 s.

FVC, Forced Vital Capacity.

*p < 0.05, **p < 0.01, ***p < 0.001 compared to Preterm No BPD.

^†^p < 0.05, ^††^p < 0.01, ^†††^p < 0.001 compared to Term-born.

^§^Antenatal corticoteroids data missing for 5 cases (4 Preterm-born No BPD, 1 Preterm-born BPD).

A total of 235 metabolites related to primary metabolism (carbohydrates and sugar phosphates, amino acids, hydroxyl acids, free fatty acids, purines, pyrimidines, aromatics, and exposome-derived chemicals) were successfully detected and identified from the BinBase database, with 128 (54.5%) having mean and median peak intensities greater than the limit of detection, and 38 (16.2%) metabolites being detected above the limit of detection in every sample analysed. Details of all detected metabolites and the number of samples in which they were present are given in Supplementary Table 1. Overall, the metabolite content of EBC was relatively low, with several metabolites close to the limit of detection in multiple samples.

### Metabolomic differences between BPD and No BPD groups

**Figure 1:**
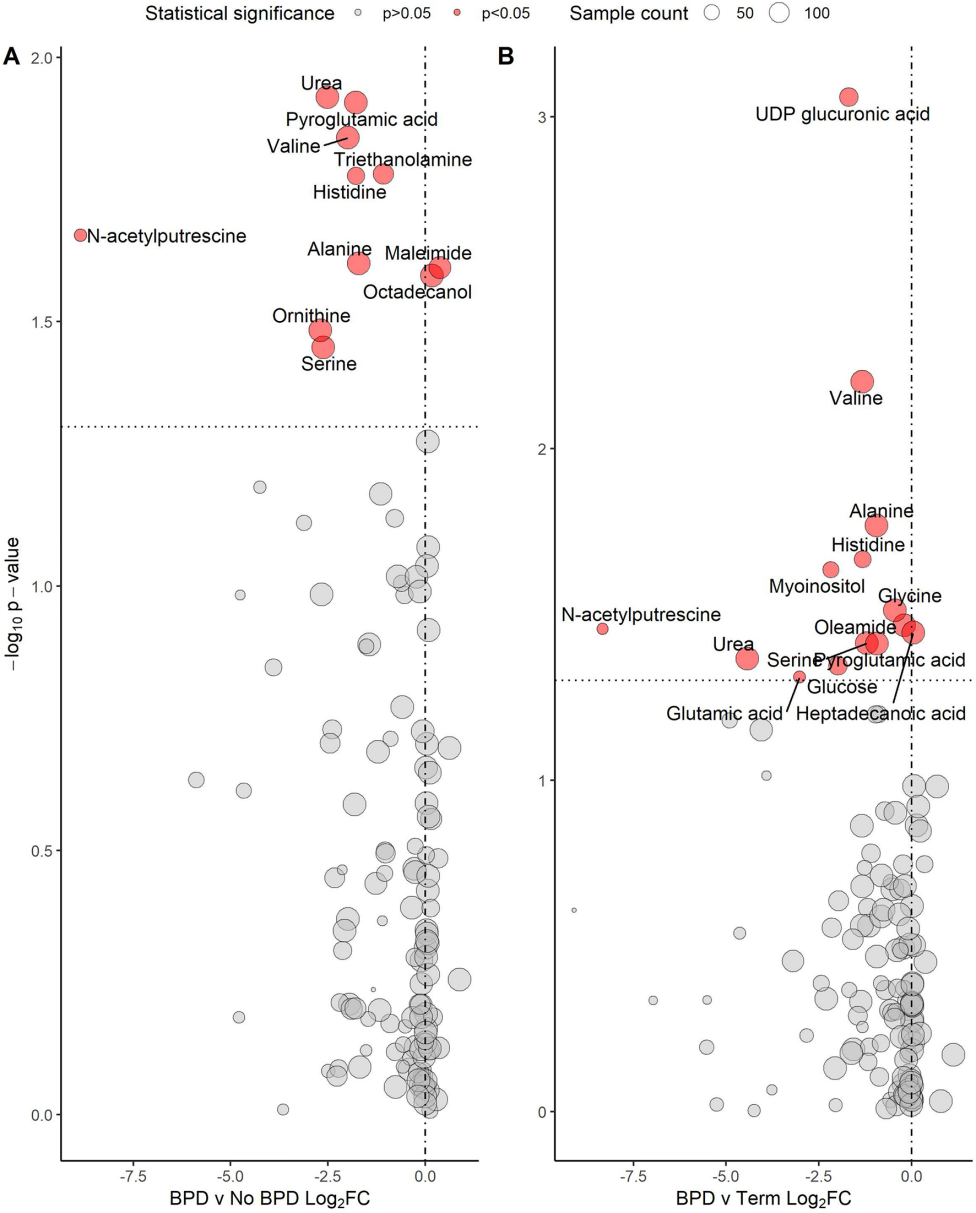
Volcano plots demonstrating significantly altered metabolites between groups (**A**) BPD vs No BPD (**B**) BPD vs Term**.** Vertical line represents a Log_2_FC of 0. Horizontal line is equivalent to p-value 0.05. Size of point is relative to number of samples in which metabolite was detected. Metabolite name given if p < 0.05. BPD, Bronchopulmonary dysplasia; Log_2_FC, log_2_ fold-change between groups.

**Figure 2:**
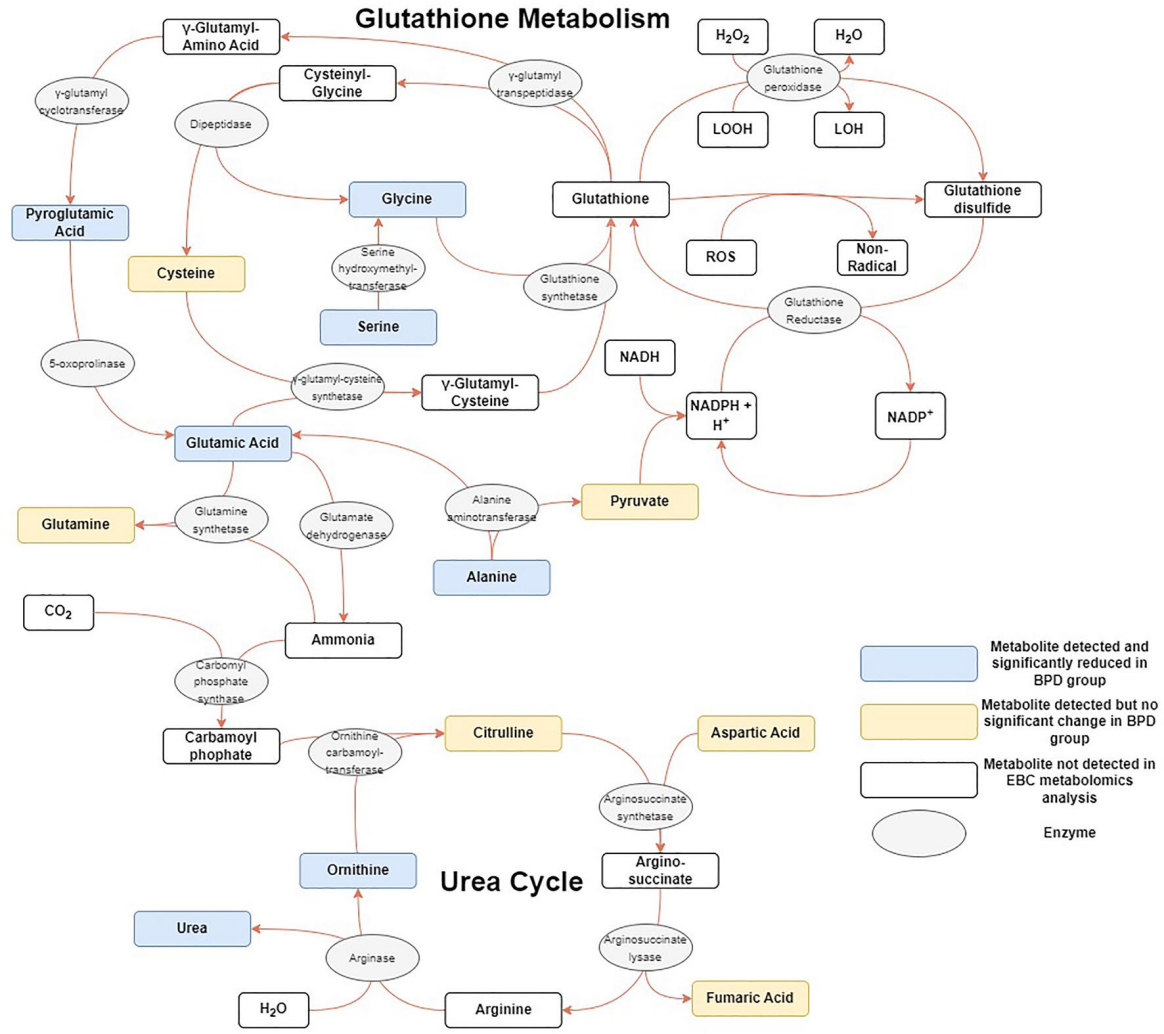
Graphic representation of glutathione metabolism and the urea cycle, highlighting metabolites detected in this analysis and those with a significantly reduced concentration in the BPD group. LOOH, lipid hydroperoxide; LOH, lipid hydroxide; ROS, reactive oxygen species; NADP, nicotinamide adenine dinucleotide phosphate; NADPH, nicotinamide adenine dinucleotide phosphate hydrogen; NADH, nicotinamide adenine dinucleotide + hydrogen.

Significant log_2_FC differences were noted between BPD and No BPD groups for ten metabolites (Fig. 1, Table 2). Alanine was reduced in the BPD group (log_2_FC − 1.71, p = 0.025) and octadecanol increased (0.17, 0.026), with both metabolites detected in every sample. Urea (− 2.52, 0.012), pyroglutamic acid (− 1.78, 0.012), valine (− 1.98, 0.014), ornithine (− 2.69, 0.033) and serine (− 2.62, 0.035) were all detected in > 98% of samples, all with a significantly lower quantity in the BPD group. MSEA (Table 3) linked alanine, ornithine and urea with a significant alteration of urea cycle metabolism (p =  < 0.001) and alanine and pyroglutamic acid with an alteration of glutathione metabolism (p = 0.008) (Fig. 2). Ornithine and urea were also significantly linked with an alteration of arginine and proline metabolism (p = 0.047).

**Table 2:** Significantly different metabolites between phenotypes.

Metabolite	Retention index	m/z	PubChem ID	% of samples	Fold change	log_2_FC	p value
**BPD vs No BPD**							
Urea	323728	189	1176	99.3	0.17	− 2.52	0.012
Pyroglutamic acid	485935	156	7405	99.3	0.29	− 1.78	0.012
Valine	313502	144	6287	99.3	0.25	− 1.98	0.014
Triethanolamine	531892	262	7618	73.6	0.47	− 1.08	0.017
Histidine	663790	154	6274	49.3	0.29	− 1.78	0.017
Alanine	244189	116	5950	100	0.31	− 1.71	0.025
Maleimide	245118	154	10935	89.9	1.30	0.37	0.025
Octadecanol	755409	327	8221	100	1.13	0.17	0.026
Ornithine	619196	142	88747248	99.3	0.15	− 2.69	0.033
Serine	395020	204	5951	98.6	0.16	− 2.62	0.035
**BPD vs Term**							
UDP-glucuronic acid	585473	217	17473	60.6	0.31	− 1.68	0.0009
Valine	313502	144	6287	99.0	0.40	− 1.33	0.006
Alanine	244189	116	5950	100	0.52	− 0.94	0.017
Histidine	663790	154	6274	48.1	0.40	− 1.31	0.022
Myoinositol	730022	305	892	42.3	0.22	− 2.17	0.023
Glycine	368707	248	750	100	0.73	− 0.45	0.031
Oleamide	849710	144	5283387	92.3	0.87	− 0.20	0.034
*N*-acetylputrescine	595523	174	122356	16.3	0.003	− 8.33	0.035
Heptadecanoic acid	751309	117	10465	100	1.04	0.06	0.036
Serine	395020	204	5951	99.0	0.43	− 1.20	0.039
Pyroglutamic acid	485935	156	7405	99.0	0.52	− 0.93	0.039
Urea	323728	189	1176	99.0	0.05	− 4.43	0.043
Glucose	659798	319	64689	59.6	0.25	− 1.98	0.045
Glutamic acid	529100	246	33032	19.2	0.12	− 3.01	0.049

BPD, Bronchopulmonary dysplasia; m/z, mass-to-charge ratio; Log_2_FC, Log_2_ fold change.

p values represent between group comparisons using t-test.

**Table 3:** Metabolite set enrichment analysis demonstrating altered biological processes implicated by significantly altered metabolite quantities.

Process	Enriched metabolites	Enrichment ratio	p value	FDR
**BPD vs No BPD**				
Urea cycle	Alanine, ornithine, urea	15.2	0.0006	0.065
Glutathione metabolism	Alanine, pyroglutamic acid	13.9	0.008	0.39
Methylhistidine metabolism	Histidine	36.6	0.027	0.89
Arginine and proline metabolism	Ornithine, urea	5.5	0.047	1.0
**BPD vs Term**				
Glutathione metabolism	Alanine, glutamic acid, glycine, pyroglutamic acid	19.5	0.00003	0.003
Glucose-alanine cycle	Alanine, glucose, glutamic acid	23.6	0.0002	0.009
Alanine metabolism	Alanine, glycine, glutamic acid	18.1	0.0004	0.014
Urea cycle	Alanine, glutamic acid, urea	10.6	0.002	0.05
Ammonia recycling	Glycine, glutamic acid, histidine	9.6	0.003	0.06
Glutamate metabolism	Alanine, glycine, glutamic acid	6.3	0.010	0.16
Arginine and proline metabolism	Glycine, urea	5.8	0.012	0.17
Glycine and serine metabolism	Glycine, glutamic acid, urea	5.2	0.016	0.20
Methylhistidine metabolism	Histidine	25.6	0.038	0.40
Beta-alanine metabolism	Glutamic acid, histidine	6.0	0.041	0.40

Univariable, unadjusted, linear regression models of the preterm-born cohort studying demographic and lung function characteristics identified that alanine (beta − 0.18, p = 0.025) and urea (− 0.29, 0.013) were only significantly associated with a history of BPD (Table 4). In univariable linear regression models for pyroglutamic acid and ornithine, female sex and history of BPD were both significantly related to a reduced quantity of these metabolites. When combined into a multivariable linear regression model, BPD remained significantly associated with a reduced quantity of pyroglutamic acid (beta − 0.24, p = 0.016) and ornithine (− 0.24, 0.039) (Table 4). No significant associations were noted between these metabolites and current lung function in univariable linear regression models with these metabolites of interest. We observed minimal significantly altered metabolites when comparing the mild and moderate/severe BPD groups (Supplementary Fig. 2).

**Table 4:** Linear regression models for preterm-born cohort.

Variable	Pyroglutamic acid			Ornithine			Alanine			Urea		
	Beta	SE	p-value	Beta	SE	p-value	Beta	SE	p-value	Beta	SE	p-value
Univariable models												
Sex (ref = Male)	− 0.20	0.09	0.020*	− 0.22	0.10	0.031*	− 0.11	0.07	0.11	− 0.14	0.10	0.16
Birthweight (z-score)	0.001	0.03	0.96	− 0.0001	0.04	0.99	− 0.03	0.03	0.26	− 0.01	0.04	0.76
IUGR (Ref = No IUGR)	− 0.09	0.11	0.45	− 0.05	0.13	0.68	0.001	0.09	0.99	− 0.03	0.13	0.81
BPD (ref = No BPD)	− 0.25	0.10	0.013*	− 0.25	0.12	0.033*	− 0.18	0.08	0.025*	− 0.29	0.11	0.013*
Age (years)	− 0.03	0.04	0.48	− 0.04	0.04	0.40	− 0.03	0.03	0.29	− 0.02	0.04	0.71
Weight (z-score)	0.05	0.04	0.17	0.04	0.04	0.40	0.02	0.03	0.53	0.03	0.04	0.42
BMI (z-score)	0.02	0.03	0.64	0.004	0.04	0.92	− 0.001	0.03	0.96	0.01	0.04	0.75
Asthma (Ref = No Asthma)	− 0.10	0.11	0.34	− 0.12	0.12	0.34	0.03	0.08	0.70	0.01	0.12	0.93
FEV_1_ (% predicted)	< 0.001	0.003	0.89	< 0.001	0.003	0.99	− 0.001	0.002	0.85	0.001	0.003	0.81
FVC (% predicted)	< 0.001	0.004	0.92	− 0.001	0.004	0.73	− 0.001	0.003	0.84	− 0.002	0.004	0.53
FEV_1_/FVC	− 0.13	0.47	0.78	− 0.08	0.55	0.89	− 0.16	0.37	0.66	0.35	0.54	0.52
FEF_25-75%_ (%predicted)	0.001	0.002	0.42	0.001	0.002	0.75	< 0.001	0.001	0.92	0.002	0.002	0.34
EBC volume collected (ml)	0.02	0.11	0.88	0.04	0.14	0.77	− 0.03	0.10	0.80	0.12	0.13	0.36
Multivariable models												
Sex (ref = Male)	− 0.20	0.08	0.021*	− 0.21	0.10	0.038*						
BPD (ref = No BPD)	− 0.24	0.10	0.016*	− 0.24	0.12	0.039*						

Univariable regression models where p < 0.1 included in multivariable model. Multivariable models not created where only one variable has a p < 0.1 in univariable models.

IUGR, intrauterine growth restriction; BPD, Bronchopulmonary Dysplasia; BMI, body mass index; FEV_1_, forced expiratory volume in 1 s; FVC, forced vital capacity; EBC, exhaled breath condensate.

* and [bold] denotes p < 0.05.

### Metabolomic differences between BPD and Term groups

Significant log_2_FC were observed between BPD and Term groups for 14 metabolites (Fig. 1, Table 2). As in the preterm-born cohort, significantly reduced quantities of valine (log_2_FC − 1.33, p = 0.006), alanine (− 0.94, 0.017), serine (− 1.2, 0.039), pyroglutamic acid (− 0.93, 0.039) and urea (− 4.43, 0.043) were seen in the BPD group when compared to term-born children. Glycine was detected in every sample, again with a significantly decreased quantity in the BPD group (− 0.45, 0.031). Oleamide was detected in > 90% of samples, with a significantly reduced quantity in the BPD group (− 0.2, 0.034). MSEA (Table 3) linked alanine, glycine and pyroglutamic acid with a significant alteration of glutathione metabolism (p =  < 0.001), and alanine, glutamic acid and urea with a significant alteration of urea cycle metabolism (p = 0.002) (Fig. 2). There was also a significant alteration of Glucose-Alanine cycle (p =  < 0.001) and Alanine metabolism (p =  < 0.001); however, glutamic acid was implicated in both processes and was detected in < 20% of samples.

**Figure 3:**
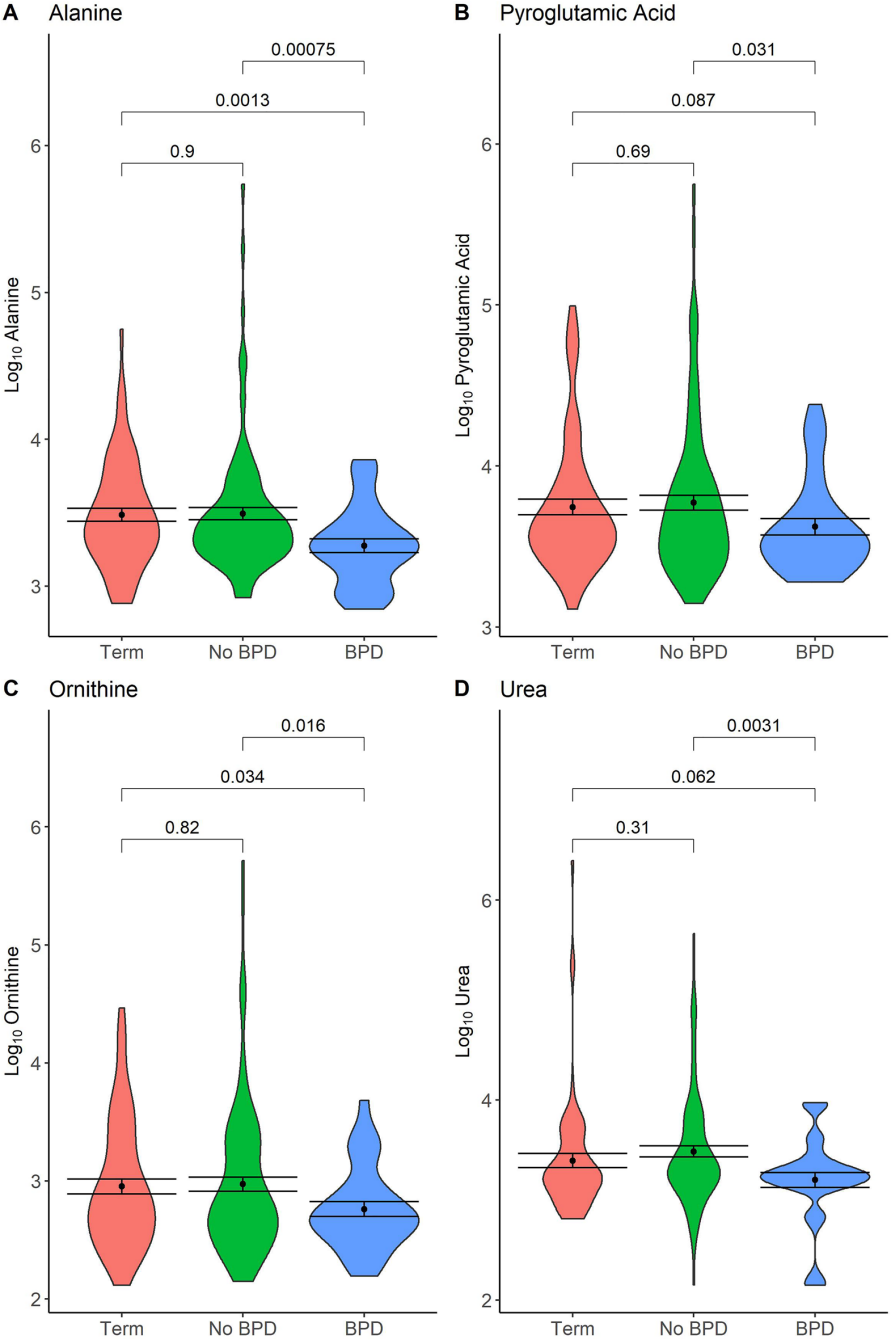
Violin Plots of Significantly Altered Metabolites in BPD group. Black dot and bars give mean and standard error of the mean (SEM). Horizontal bars give p-values from ANOVA with post-hoc Bonferroni correction for between group comparisons.

Figure 3 shows results for ANVOA with post-hoc Bonferroni comparisons between BPD, No BPD and Term groups for alanine, pyroglutamic acid, ornithine and urea. All four metabolites showed a consistent trend of the lowest quantities being present in the BPD group, with both alanine and ornithine having a significantly lower quantity in the BPD group when compared to both the No BPD (p =  < 0.001, p = 0.016 respectively) and Term (0.0013, 0.034 respectively) groups. Pyroglutamic acid and urea had a significantly lower quantity in the BPD group compared to No BPD (0.031, 0.0031 respectively), with a near-significant difference when compared to the Term group (0.087, 0.062 respectively).

## Discussion

In this exploratory study of the EBC metabolome of preterm-born school-aged children, we have demonstrated significant differences in several metabolites from those with a history of BPD. On comparison to both preterm-born and term-born controls, levels of alanine, pyroglutamic acid, serine, urea and valine were all significantly lower in the BPD group. These five metabolites were detected in > 98% of samples. Alanine and pyroglutamic acid were significantly associated with an alteration of glutathione metabolism. Alanine and urea were significantly associated with an alteration in the urea cycle (with ornithine also being associated when compared to preterm-born controls). Linear regression analyses demonstrated that alanine, pyroglutamic acid, ornithine and urea remained significantly associated with BPD in the preterm-born group when other participant characteristics were also considered. However, linear regression analyses did not show a significant association between any of these metabolites and current lung function parameters.

Pyroglutamic acid, also known as 5-oxoproline, is an intermediary in glutathione synthesis and recycling. Glutathione is a potent antioxidant, and under conditions of oxidative stress, where glutathione is consumed, pyroglutamic acid levels also become low^28^. Alanine concentration also appears to be reduced in metabolomic studies of murine models of pulmonary inflammation, along with pyroglutamic acid^29^. Alanine is a non-essential amino acid that is a constituent of nearly all proteins. Whilst it is not a direct precursor to glutathione, alanine can be converted to pyruvate, a key intermediate of glucose metabolism^30^. Glucose metabolism is an important source of reducing substances, such as NADPH, which are essential in glutathione synthesis^31, 32^. Alanine can also be converted to other amino acids, such as serine, which is a precursor to glycine^32^. Glycine is a key, rate-limiting amino acid for glutathione synthesis. Glutathione consumption increases metabolism of glycine, as well as inflammatory conditions reducing glycine availability^31, 32^. We noted a significant decrease in serine in our BPD group when compared with the No BPD group, as well as a significant reduction in glycine when compared to the Term group. Taken together, the metabolomic differences observed in the BPD group suggest decreased glutathione levels, and thereby suggesting persistent oxidative stress, in the airways of preterm-born children with a history of BPD.

Glutathione has previously been shown to provide first-line defense against pulmonary oxidative injury. Adult studies have shown that glutathione concentrations in the airway’s ELF are many times greater than those seen in plasma^33^, and animal models have shown that pulmonary glutathione depletion enhances oxidant toxicity^34^. In the paediatric population, alterations of glutathione metabolism have been linked with respiratory pathology. A study of ELF in children with severe asthma reported significantly decreased concentration of glutathione, with evidence of glutathione consumption by oxidative stress, further supported by increased levels of hydrogen peroxide (H_2_O_2_), a powerful oxidant. However, there was no significant association between markers of impaired glutathione metabolism and FEV_1_^35^. Impaired glutathione metabolism has also been associated with impaired macrophage function in the airways of children with severe asthma, with glutathione supplementation restoring macrophage function^36^. Reduced concentration of glutathione secondary to oxidative stress has also been described in bronchoalveolar lavage fluid (BAL) obtained from children with cystic fibrosis, with those experiencing an infective exacerbation having a further decreased concentration^37^.

Previous mechanistic studies of BPD in later childhood and adulthood have implicated cytotoxic CD8 + T-lymphoytes^38^, elevated neutrophils and pro-inflammatory cytokines^39^, and thickened basement membranes and airway remodeling^40^. However, to our knowledge, this is the first study to analyze the EBC metabolome of preterm-born children with a history of BPD. Previous studies in preterm infants have linked increases in pulmonary oxidative stress and glutathione metabolism to the development of BPD. In a small study of BAL from preterm infants born < 34 weeks’ gestation, lower glutathione levels on the first day of life were associated with increased development of BPD at 36 weeks’ gestation^41^. This study was performed before the routine use of surfactant replacement therapy. A further study in the post-surfactant era of infants born at < 32 weeks’ gestation reported lower BAL glutathione levels in the first 24 h of life in those who later developed BPD, lower glutathione levels in those who had delayed surfactant administration, and higher concentrations of malondialdehyde, suggestive of oxidative damage, in the BPD group^42^. Animal models have further supported the role of glutathione in lung injury, with glutathione deficient mice having impaired tolerance of oxidative stress, and abnormal early lung development^43^. Whilst glutathione metabolism has been implicated in our study by MSEA, we did not detect glutathione in either its reduced or oxidized form. However, glutathione has a short half-life of approximately ten minutes, which can make it’s detection challenging^44^. One previous study has examined oxidative stress in the airways of preterm-born adolescents, measuring 8-isoprostane (a product of lipid peroxidation in the presence of oxygen free radicals) in EBC, both with and without BPD. These individuals, born during the peri-surfactant era, demonstrated evidence of persistent airway oxidative stress, with increased 8-isoprostane when compared to term-born controls^45^. However, in this study no significant difference was observed for 8-isoprostane between BPD and No BPD groups, and no correlation was seen between 8-isoprostane levels and spirometry values.

Previous metabolomic studies of BPD, with similar analytical techniques to our study, have focused on preterm-born infants in the neonatal period. A study of tracheal aspirates taken from infants born < 30 weeks’ gestation using metabolomic techniques reported that 19 metabolites discriminated infants who subsequently did or did not develop BPD^14^, including alterations in amino acids (citrulline and symmetric dimehtlyarginine) involved in nitric oxide metabolism, as well as an increase in serine. The authors also observed increased acylcarnitines which are released after β-oxidation of fatty acids during oxidative stress. In contrast, another metabolomic study of tracheal aspirates from infants ≤ 28 weeks’ gestation noted that early decreases in fatty-acid metabolism, particularly the fatty acid β-oxidation pathway, may predispose infants to developing BPD^46^. A nuclear magnetic resonance metabolomics study of urine from 18 infants born < 28 weeks’ gestation showed decreased glycine levels in those who subsequently developed BPD, similarly to our study, also suggesting impaired glutathione metabolism. The authors also found increased alanine in the BPD group, which they attributed to increased cellular metabolism demands, due to an inflammatory process^47^. These authors studied infants in the first week of life, where the pathophysiology is respiratory distress syndrome and pulmonary surfactant deficiency, as opposed to the chronic inflammation seen in BPD, potentially explaining the different alanine levels we observed in our study.

In our study, we observed significant decreases of alanine, ornithine, and urea in the BPD group, with MSEA linking these changes to a significant decrease in urea cycle metabolism. The urea cycle removes ammonia, produced during protein catabolism, preventing cellular toxicity. Animal models have demonstrated increased ammonia levels lead to intracellular production of reactive oxygen species, induction of cellular apoptosis in bovine epithelial cells, increased inflammatory cytokines and repression of DNA repair-related genes in porcine Type II alveolar epithelial cells^48, 49^. We also observed a significant reduction in histidine in our BPD group when compared to both the No BPD and Term groups. Histidine, an essential amino acid, is metabolised to histamine and can affect the contractility of bronchial smooth muscle and cause airway oedema^50^. Histidine itself has also been reported to have antioxidant properties, being a scavenger of free hydroxyl and singlet oxygen radicals and inhibiting fatty-acid oxidation during in vitro studies^51^. Lower quantities of urea and ornithine in the BPD group were also significantly linked to arginine and proline metabolism. Arginine is metabolized into either nitric oxide by nitric oxide synthetase (NOS) or into urea and ornithine by arginase. Increased arginase activity is thought to play a role in childhood asthma pathogenesis, with a consequent increase in proline production leading to collagen deposition^52^. Whilst elevated FE_NO_ is used clinically as a biomarker of asthma^53^, methylated products of arginine can inhibit NOS activity and contribute to airway oxidative stress in specific phenotypes of asthma associated with obesity^54^. The reduced quantities of metabolites linked to protection from reactive oxidant species in those with BPD implied by our results suggest a persistent deficit since the neonatal period, especially for glutathione, however, this is speculative. We did not observe any associations between metabolic processes involved with oxidative stress and current lung function. This may be due to other processes such as functional and structural abnormalities also significantly contributing to the development of PLD as we have recently reported^55, 56^. However, one previous study using superoxide dismutase did not decrease rates of BPD in the neonatal period but was associated with decreased respiratory symptoms at one year of age^57^. Whether such treatments are beneficial for PLD will require further study. In addition, longitudinal metabolome analysis may reveal emerging mechanisms related to lung function decline.

This study represents one of the largest metabolomic studies of a clinical cohort. The significantly altered metabolites of interest in the BPD group were detected in all or nearly all samples analyzed, ensuring robust results. Using an untargeted metabolomics approach, we have been able to identify patterns of changes in multiple metabolites which we have been able to link with biological processes. We have studied a cohort of children who had experienced contemporary standard of neonatal care, and by using EBC we have been able to directly sample ELF, which is representative of airway biochemistry in a simple and well-tolerated manner. Although EBC volumes varied between our clinical groups, linear regression analyses did not reveal a significant association between metabolite quantities and EBC volume. As the overall metabolite content of EBC was low, as has been previously reported^58^, there may have been metabolites present that were below the limit of detection for the mass spectrometry method used. We combined our mild and moderate/severe BPD groups as few differences were noted for metabolites between these two groups thus it was reasonable to combine these two groups. Dietary intake has recently been shown to modulate the breath metabolome^59^, however we had insufficient nutritional data to adjust for this potential confounder. Similarly, whilst data was initially collected on antenatal and household smoking, overall rates in the preterm-born cohort were low suggesting high recall bias, and therefore were not included in our analyses. Whilst a reference metabolite would be useful to normalize metabolite concentrations, as in urine metabolomics^60^, this is currently not possible with EBC samples^61^. Ideally, our findings should be replicated using a validation cohort, however we are limited by the number of large contemporaneous cohorts available to study.

In conclusion, this exploratory mass spectrometry-based analysis of the EBC metabolome has revealed significant reductions in metabolites related to antioxidant pathways in the airways of preterm-born school-aged children with a history of BPD, many years after the initial pulmonary insult. Future work should focus on therapeutic strategies to improve antioxidant mechanisms in these children, with a possible improvement in longer-term respiratory outcomes.

### Supplementary Information

Supplementary Information.

## Data Availability

Data from the RHiNO study is available to research collaborators subject to confidentiality and non-disclosure agreements. Contact Professor Sailesh Kotecha (kotechas@cardiff.ac.uk) for any data requests.
