# Supplementary material for: Evidence of abnormality in glutathione metabolism in the airways of preterm born children with a history of bronchopulmonary dysplasia
Source: Sci Rep. 2023 Nov 9;13:19465. doi: 10.1038/s41598-023-46499-w (PMC10636015; doi:10.1038/s41598-023-46499-w)
Supplement: Supplementary file 1 — Supplementary Information. [file 41598_2023_46499_MOESM1_ESM.pdf]

Evidence of abnormality in glutathione metabolism in the airways of preterm born children with a history of BPD – Online Supplement

Christopher W Course MBBCh[1], Philip A Lewis PhD[2], Sarah J Kotecha PhD[1], Michael Cousins PhD[1,3], Kylie Hart PhD[3], Kate J Heesom PhD[2], W John Watkins PhD[1], Sailesh Kotecha PhD[1].

[1]Department of Child Health, School of Medicine, Cardiff University, Cardiff, United Kingdom.

[2]Faculty of Life Sciences, University of Bristol, Bristol, United Kingdom

[3]Department of Paediatrics, Cardiff and Vale University Health Board, Cardiff, United Kingdom

Corresponding Author: Professor Sailesh Kotecha

Department of Child Health

Cardiff University School of Medicine

Heath Park

Cardiff CF14 4XN

United Kingdom

kotechas@cardiff.ac.uk

Supplementary Figure 1: Flow diagram describing recruitment to the RHiNO study and EBC analysis.

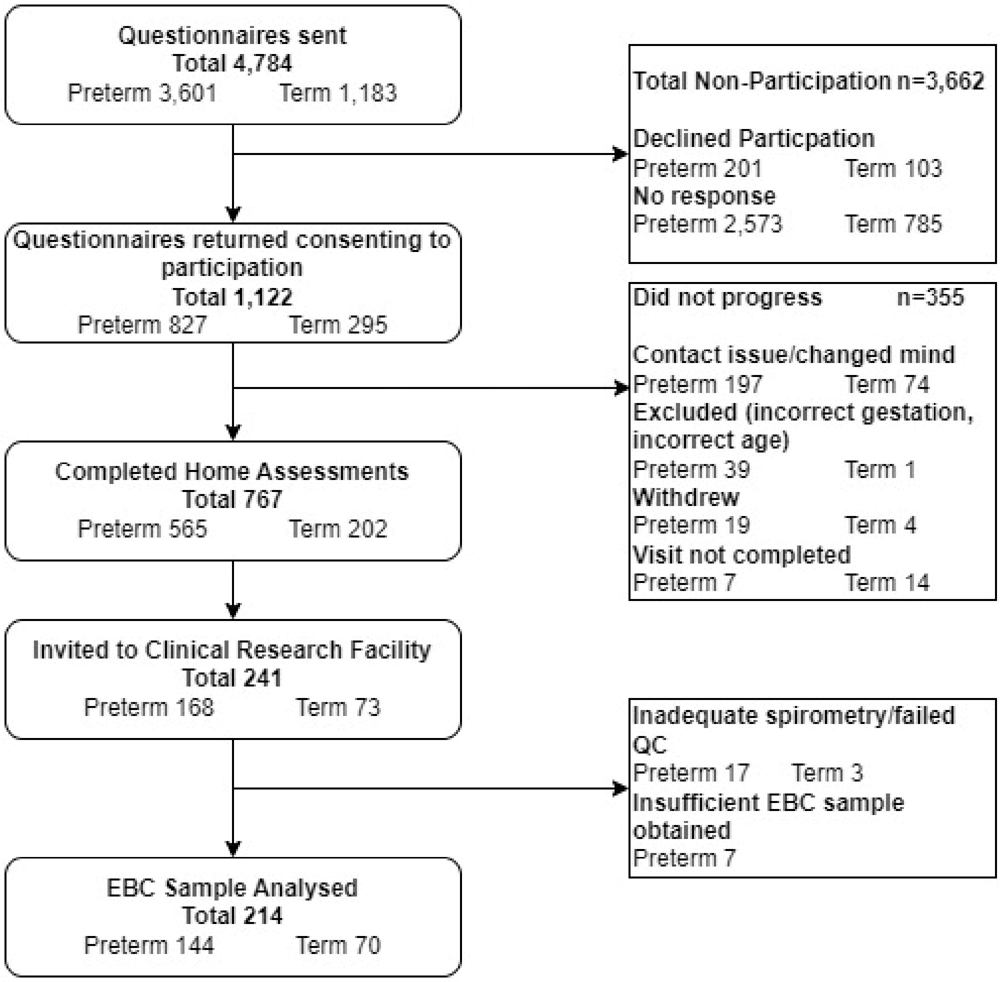

Supplementary Figure 2: Volcano plot showing significantly altered proteins between the mild and moderate/severe BPD groups. Vertical line represents a Log_2FC of 0. Horizontal line is equivalent to p-value 0.05. Size of point is relative to number of samples in which metabolite was detected. Metabolite name given if $p<0.05$. BPD: Bronchopulmonary dysplasia; Log_2FC: Log_2 fold-change between groups.

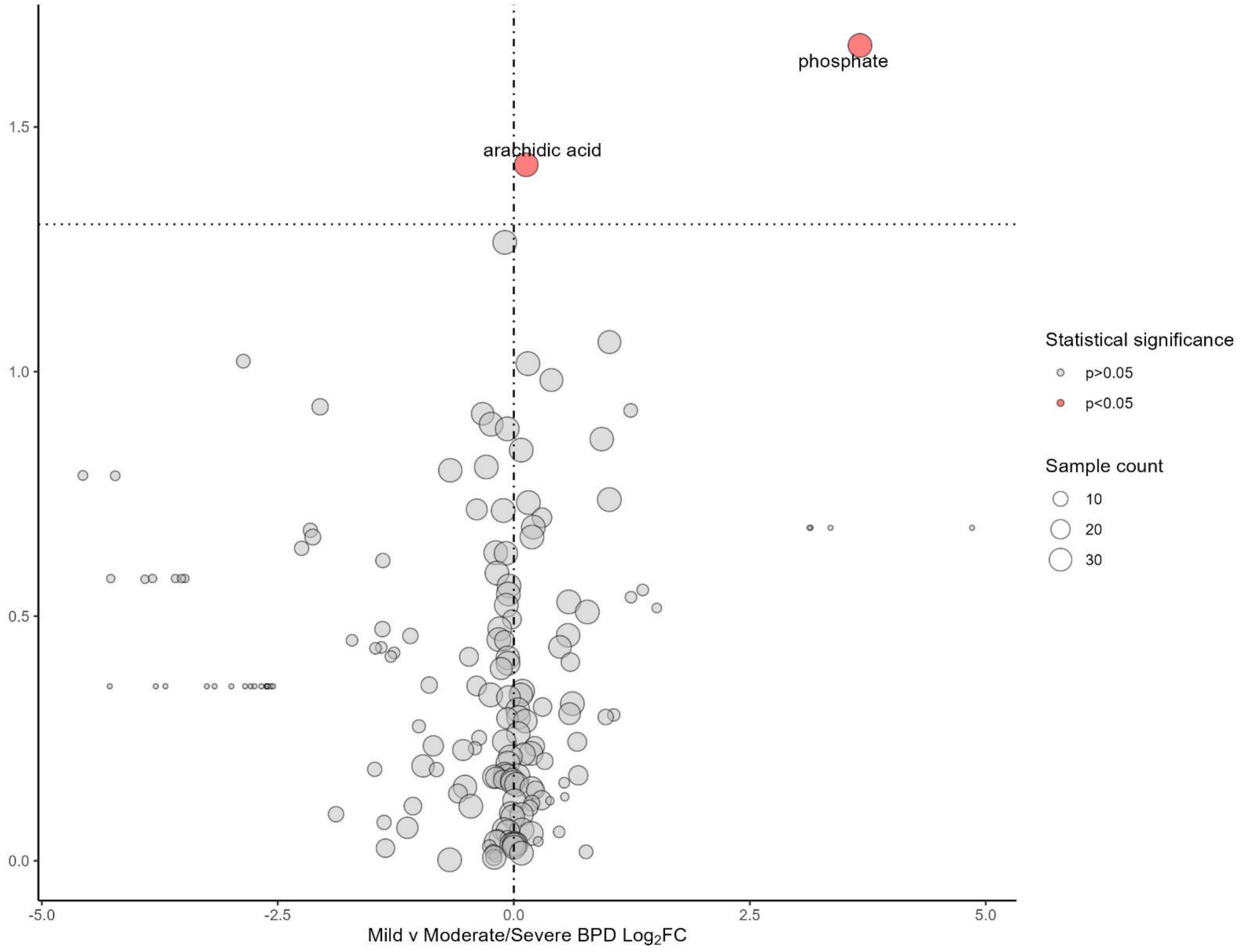

Supplementary Table 1: List of all detected metabolites, percentage of samples in which they were detected and mean and standard deviation of each metabolite by group (data log_{10} transformed).

Metabolite	% BPD Samples	BPD mean	BPD SD	% NoBPD Samples	NoBPD mean	NoBPD SD	% Term samples	Term mean	Term SD
1,2,4-benzenetriol	23.5	1.81	0.29	30.9	1.88	0.29	29.6	1.78	0.36
1,2-anhydromyoinositol	5.9	1.33	0.53	14.5	1.63	0.42	9.9	1.58	0.35
1-hexadecanol	100	2.50	0.11	99.1	2.48	0.14	100	2.48	0.12
1-kestose	0	1.48	0.20	4.5	1.51	0.30	1.4	1.50	0.29
1-methyladenosine	0	1.40	0.22	0.9	1.42	0.23	1.4	1.37	0.26
1-monostearin	100	2.73	07	99.1	2.75	07	100	2.72	0.10
2,3-bisphosphoglyceric acid	26.5	1.86	0.33	35.5	1.96	0.50	29.6	1.92	0.53
2,3-dihydroxybutanoic acid	0	1.45	0.23	0	1.44	0.29	2.8	1.44	0.33
2,8-dihydroxyquinoline	0	1.49	0.14	0	1.39	0.22	1.4	1.45	0.19
2-aminophenol	0	1.51	0.23	0	1.55	0.20	1.4	1.50	0.23
2-deoxytetronic acid	5.9	1.70	0.18	5.5	1.74	0.21	11.3	1.78	0.34
2-hydroxy-2-methylbutanoic acid	97.1	3.08	0.68	91.8	3.16	0.66	93.0	3.13	0.59
2-hydroxyglutaric acid	35.3	1.95	0.13	30.9	1.93	0.16	42.3	1.99	0.18
2-hydroxyhippuric acid	2.9	1.50	0.31	0	1.44	0.23	1.4	1.38	0.35
2-hydroxypyrazinyl-2-propenoic acidethylester	100	3.55	08	99.1	3.53	0.13	100	3.57	0.10
2-hydroxyvaleric acid	100	3.50	04	99.1	3.45	0.17	100	3.49	06
2-ketoisocaproic acid	100	3.04	0.31	98.2	3.07	0.33	98.6	3.11	0.31
2-methylglyceric acid	0	1.55	0.18	2.7	1.57	0.27	4.2	1.64	0.23
2-monopalmitin	100	2.98	0.55	99.1	2.90	0.57	100	2.81	0.47
2-picolinic acid	17.6	1.75	0.38	35.5	1.81	0.39	36.6	1.83	0.46
3,3-hydroxyphenyl-3-hydroxypropionic acid	0	1.50	0.23	1.8	1.49	0.22	4.2	1.53	0.38
3,3-hydroxyphenylpropionic acid	100	2.49	04	98.2	2.48	0.11	100	2.49	03
3,4-dihydroxybenzoic acid	0	1.71	0.18	3.6	1.70	0.22	5.6	1.69	0.36
3,4-dihydroxycinnamic acid	55.9	2.03	0.13	60.9	2.03	0.20	63.4	2.06	0.18
3,4-dihydroxyhydrocinnamic acid	8.8	1.62	0.27	16.4	1.72	0.43	12.7	1.70	0.29
3,4-dihydroxyphenylacetic acid	20.6	1.68	0.44	19.1	1.63	0.40	21.1	1.67	0.40
3,6-anhydro-D-galactose	2.9	1.47	0.28	0	1.44	0.26	1.4	1.51	0.30
3-aminoisobutyric acid	73.5	2.19	0.24	67.3	2.16	0.32	78.9	2.23	0.29
3-hydroxy-3,4-hydroxy-3-methoxyphenylpropionic acid	0	1.44	0.22	0	1.42	0.32	1.4	1.44	0.35
3-hydroxy-3-methylglutaric acid	0	1.46	0.18	0	1.41	0.27	1.4	1.49	0.27
3-hydroxyphenylacetic acid	0	1.48	0.20	0	1.50	0.18	2.8	1.54	0.27
3-hydroxypropionic acid	100	3.00	06	99.1	3.00	0.11	100	3.00	07
3-phosphoglycerate	0	1.48	0.17	1.8	1.44	0.25	1.4	1.50	0.24
4-hydroxybenzoate	44.1	2.00	0.21	47.3	1.99	0.20	52.1	2.00	0.27
4-hydroxyhippuric acid	14.7	1.65	0.40	20.9	1.69	0.37	28.2	1.81	0.38
4-hydroxyphenylacetic acid	2.9	1.45	0.37	5.5	1.48	0.36	5.6	1.53	0.41
4-methylcatechol	0	1.52	0.18	0	1.51	0.19	1.4	1.49	0.22
4-pyridoxic acid	0	1.37	0.24	0	1.40	0.28	1.4	1.43	0.28
5-aminovaleric acid	23.5	1.93	0.17	35.5	1.98	0.31	22.5	1.92	0.18

5-deoxy-5-methylthioadenosine	0	1.38	0.29	0	1.38	0.34	1.4	1.47	0.28
5-hydroxy-3-indoleacetic acid	0	1.39	0.32	0	1.42	0.20	1.4	1.42	0.29
5-hydroxymethyl-2-furoic acid	100	2.29	0.10	98.2	2.27	0.10	100	2.29	0.16
6-deoxyglucitol	0	1.45	0.25	0.9	1.45	0.26	1.4	1.44	0.29
7-methylguanine	0	1.41	0.32	0	1.44	0.27	1.4	1.45	0.28
aconitic acid	0	1.50	0.27	3.6	1.54	0.26	4.2	1.61	0.38
adenine	0	1.47	0.23	0	1.48	0.21	1.4	1.48	0.25
adenosine	2.9	1.53	0.25	1.8	1.55	0.39	7.0	1.58	0.40
adipic acid	100	3.28	0.23	99.1	3.27	0.21	100	3.26	0.18
alanine	100	3.25	0.27	99.1	3.42	0.43	100	3.42	0.36
allantoic acid	0	1.47	0.20	0	1.45	0.26	1.4	1.42	0.36
alloxanoic acid	8.8	1.84	0.15	15.5	1.83	0.21	11.3	1.88	0.15
alphaketoglutarate	0	1.47	0.26	4.5	1.51	0.40	7.0	1.53	0.43
aminomalonate	2.9	1.45	0.39	10	1.62	0.33	7.0	1.59	0.29
anthranilic acid	0	1.43	0.23	0	1.47	0.30	2.8	1.53	0.32
arabitol	100	2.77	07	99.1	2.80	0.11	100	2.83	0.27
arachidic acid	100	3.55	05	99.1	3.56	0.12	100	3.55	0.10
ascorbic acid	0	1.41	0.20	0	1.34	0.27	1.4	1.45	0.22
asparagine	100	2.70	08	99.1	2.73	0.18	100	2.73	0.17
aspartic acid	100	2.70	0.12	99.1	2.78	0.40	100	2.77	0.28
azelaic acid	97.1	2.32	0.14	96.4	2.33	0.17	93.0	2.30	0.22
benzoic acid	100	4.10	04	99.1	4.10	04	100	4.10	04
betaalanine	0	1.40	0.35	3.6	1.53	0.25	1.4	1.48	0.29
betagentiobiose	14.7	1.71	0.24	19.1	1.71	0.45	15.5	1.63	0.46
betamannosylglycerate	0	1.39	0.32	0	1.44	0.31	1.4	1.43	0.33
biphenyl	100	2.34	06	99.1	2.33	07	100	2.32	07
butane-2,3-diol	100	3.50	0.32	99.1	3.61	0.48	100	3.59	0.41
butyrolactam	88.2	2.19	0.47	89.1	2.28	0.35	90.1	2.24	0.37
capric acid	100	3.17	07	99.1	3.16	07	100	3.16	06
caprylic acid	100	3.91	04	99.1	3.89	05	100	3.91	05
catechol	0	1.53	0.22	0	1.51	0.26	2.8	1.43	0.36
cellobiose	79.4	2.25	0.32	81.8	2.31	0.48	77.5	2.28	0.40
cerotinic acid	91.2	2.20	0.12	91.8	2.20	0.13	90.1	2.18	0.17
cholesterol	61.8	2.11	0.23	68.2	2.16	0.35	57.7	2.09	0.38
citramalic acid	14.7	1.74	0.21	14.5	1.75	0.34	14.1	1.76	0.29
citric acid	91.2	2.38	0.29	96.4	2.49	0.44	97.2	2.54	0.58
citrulline	14.7	1.73	0.32	16.4	1.74	0.41	18.3	1.80	0.40
conduritolbetaepoxide	26.5	1.71	0.47	22.7	1.74	0.50	38.0	1.92	0.55
creatinine	29.4	1.77	0.73	30.9	1.89	0.76	25.4	1.83	0.95
cystathionine	0	1.51	0.27	3.6	1.51	0.37	2.8	1.56	0.30
cysteine	2.9	1.55	0.36	10.9	1.60	0.38	12.7	1.64	0.48
cysteineglycine	32.4	1.84	0.41	42.7	1.91	0.43	42.3	1.96	0.39
cystine	26.5	1.84	0.22	20.9	1.86	0.20	42.3	1.94	0.23
dehydroabietic acid	97.1	3.00	0.43	96.4	2.91	0.49	95.8	2.87	0.56
dehydroascorbic acid	58.8	2.00	08	49.1	1.98	0.15	50.7	2.02	0.20
deoxypentitol	0	1.48	0.16	5.5	1.54	0.30	4.2	1.55	0.35
digalacturonic acid	29.4	1.92	0.12	30	1.92	0.14	39.4	1.93	0.15
digitoxose	100	2.60	0.13	99.1	2.57	0.17	100	2.64	0.15

diglycerol	100	2.73	06	99.1	2.73	08	100	2.74	0.13
dihydro-3-coumaric acid	100	2.51	05	97.3	2.47	0.12	100	2.50	05
docosenoic acid	100	3.27	0.42	99.1	3.32	0.58	100	3.36	0.49
dodecanol	100	3.25	0.10	99.1	3.24	0.12	100	3.25	09
enolpyruvate	2.9	1.70	0.17	2.7	1.68	0.21	0	1.68	0.18
erythritol	23.5	1.70	0.34	26.4	1.67	0.46	19.7	1.71	0.60
erythronic acid lactone	0	1.44	0.31	0	1.47	0.21	1.4	1.45	0.25
erythrose major	2.9	1.47	0.31	10.9	1.62	0.31	7.0	1.55	0.30
ethanolamine	100	2.84	0.27	96.4	2.91	0.40	98.6	2.89	0.46
ferulic acid	0	1.44	0.22	0	1.41	0.28	1.4	1.43	0.27
fructose	76.5	2.27	0.37	72.7	2.37	0.62	80.3	2.40	0.54
fucose	100	2.85	06	99.1	2.84	08	100	2.85	0.15
fumaric acid	76.5	2.13	0.15	72.7	2.13	0.24	74.6	2.14	0.27
furoylglycine	55.9	1.95	0.53	43.6	1.98	0.45	42.3	1.84	0.56
galactinol	38.2	1.98	0.31	55.5	2.08	0.34	56.3	2.10	0.37
galactitol	38.2	1.77	0.50	48.2	2.08	0.68	42.3	1.99	0.57
galactonic acid	5.9	1.47	0.32	5.5	1.54	0.28	5.6	1.56	0.38
galactose	85.3	2.37	0.39	80.9	2.54	0.62	87.3	2.53	0.62
glucoheptulose	0	1.40	0.36	0	1.44	0.30	1.4	1.49	0.32
gluconic acid	2.9	1.53	0.21	8.2	1.60	0.33	14.1	1.66	0.40
gluconic acidlactone	0	1.46	0.25	0	1.41	0.30	4.2	1.48	0.31
glucose	47.1	2.12	0.46	54.5	2.25	0.53	66.2	2.36	0.57
glucuronic acid	0	1.57	0.30	4.5	1.62	0.25	5.6	1.62	0.37
glutamic acid	8.8	1.61	0.28	20.9	1.82	0.51	25.4	1.79	0.41
glutamine	100	3.98	0.38	99.1	4.09	0.29	100	4.01	0.38
glutaric acid	88.2	2.18	0.18	79.1	2.15	0.19	84.5	2.17	0.20
glycerol	100	3.92	0.17	99.1	3.96	0.21	100	3.87	0.15
glycerol-3-galactoside	14.7	1.50	0.48	15.5	1.64	0.32	28.2	1.80	0.40
glycerol alpha phosphate	0	1.47	0.20	0	1.46	0.18	1.4	1.44	0.29
glycine	100	3.42	0.33	99.1	3.53	0.35	100	3.55	0.27
glycolic acid	100	2.82	0.16	98.2	2.83	0.20	100	2.86	0.23
glycylproline	2.9	1.53	0.22	7.3	1.59	0.31	7.0	1.61	0.41
guanine	0	1.53	0.20	0	1.50	0.22	1.4	1.47	0.27
heptadecanoic acid	100	3.73	04	99.1	3.72	04	100	3.72	03
hippuric acid	5.9	1.60	0.23	10.9	1.67	0.39	8.5	1.71	0.53
histidine	32.4	1.97	0.14	53.6	2.07	0.30	56.3	2.05	0.24
homocystine	0	1.42	0.34	3.6	1.56	0.31	4.2	1.45	0.44
homovanillic acid	5.9	1.51	0.36	6.4	1.53	0.32	7.0	1.53	0.48
hydroxycarbamate	100	2.73	0.11	99.1	2.71	0.10	100	2.70	09
hydroxyproline dipeptide	2.9	1.68	0.16	4.5	1.65	0.23	2.8	1.72	0.37
hypoxanthine	0	1.38	0.32	2.7	1.46	0.33	2.8	1.43	0.45
indole-3-acetate	0	1.52	0.20	0.9	1.45	0.25	2.8	1.56	0.32
indoxylsulfate	0	1.37	0.35	4.5	1.41	0.32	4.2	1.45	0.45
isocitric acid	0	1.41	0.18	1.8	1.50	0.24	5.6	1.54	0.43
isoleucine	100	2.86	0.14	99.1	2.96	0.36	100	2.93	0.24
isopropyl benzene	100	3.75	0.51	99.1	3.82	0.47	100	3.66	0.48
isothreonic acid	5.9	1.75	0.14	10	1.78	0.19	8.5	1.83	0.37
itaconic acid	97.1	2.69	0.32	95.5	2.56	0.28	95.8	2.67	0.50
kynurenic acid	0	1.40	0.35	0	1.47	0.21	2.8	1.42	0.38

lactic acid	100	3.62	0.24	99.1	3.69	0.50	100	3.70	0.35
lactose	35.3	1.93	0.23	38.2	1.97	0.25	43.7	1.94	0.21
lactulose	55.9	2.09	0.33	50	2.14	0.54	46.5	2.11	0.46
lauric acid	100	3.98	05	99.1	3.97	05	100	3.97	0.10
leucine	100	3.48	0.83	99.1	3.39	0.73	100	3.46	0.71
levoglucosan	32.4	1.92	0.26	47.3	1.98	0.26	39.4	2.01	0.34
levoinositol	0	1.38	0.23	0	1.37	0.34	1.4	1.36	0.42
lysine	2.9	1.59	0.18	11.8	1.67	0.47	12.7	1.65	0.39
maleimide	100	2.43	0.28	85.5	2.33	0.30	93.0	2.38	0.29
malic acid	35.3	1.99	0.35	41.8	2.08	0.45	54.9	2.12	0.40
maltose-1	91.2	2.30	0.30	90.9	2.42	0.45	84.5	2.30	0.37
mannose	23.5	1.91	0.52	37.3	2.14	0.84	42.3	2.16	0.83
metanephrine	0	1.45	0.20	0.9	1.43	0.28	1.4	1.42	0.32
methanolphosphate	26.5	1.94	0.97	23.6	1.92	0.93	40.8	2.18	1.12
methionine	14.7	1.38	0.52	12.7	1.48	0.60	12.7	1.57	0.60
methylmaleic acid	0	1.62	0.17	0.9	1.62	0.16	1.4	1.62	0.16
montanic acid	100	2.46	0.10	97.3	2.42	0.13	100	2.46	09
myoinositol	26.5	1.92	0.33	37.3	1.98	0.43	50.7	2.05	0.47
myristic acid	100	3.41	04	99.1	3.40	05	100	3.40	04
N-acetylaspartic acid	2.9	1.59	0.24	3.6	1.62	0.32	8.5	1.63	0.40
N-acetylmannosamine	2.9	1.75	0.14	4.5	1.75	0.17	5.6	1.77	0.22
N-acetylputrescine	5.9	1.59	0.26	21.8	1.86	0.74	22.5	1.74	0.67
N-carbamoylaspartate	2.9	1.44	0.20	3.6	1.51	0.28	4.2	1.54	0.29
N-carbamylglutamate	0	1.47	0.31	0.9	1.51	0.24	4.2	1.52	0.34
nepsilontrimethyllysine	0	1.67	0.20	1.8	1.64	0.28	4.2	1.64	0.22
nicotinic acid	14.7	1.76	0.25	14.5	1.80	0.54	22.5	1.84	0.49
N-methylglutamic acid	88.2	2.30	0.26	89.1	2.29	0.24	91.5	2.28	0.28
nonadecanoic acid	100	2.74	06	99.1	2.71	07	100	2.71	07
noradrenaline	0	1.50	0.29	2.7	1.53	0.33	2.8	1.46	0.28
octadecanol	100	3.05	0.12	99.1	3.01	08	100	3.02	0.10
oleamide	82.4	2.48	0.39	83.6	2.38	0.34	97.2	2.54	0.32
oleic acid	97.1	2.56	0.18	99.1	2.56	0.14	100	2.57	0.11
ornithine	97.1	2.72	0.41	99.1	2.92	0.59	97.2	2.88	0.53
orotic acid	0	1.50	0.26	0	1.37	0.34	1.4	1.43	0.35
palatinitol	0	1.42	0.32	6.4	1.49	0.38	2.8	1.52	0.28
palmitoleic acid	5.9	1.67	0.19	0	1.59	0.18	1.4	1.56	0.26
parabanic acid	94.1	2.76	0.35	94.5	2.81	0.34	95.8	2.80	0.32
pcresol	100	2.53	0.15	97.3	2.55	0.19	98.6	2.59	0.31
pentitol	0	1.34	0.21	0	1.36	0.31	1.4	1.43	0.36
pentose	73.5	2.25	0.29	65.5	2.15	0.30	60.6	2.14	0.39
phenol	100	3.20	05	99.1	3.21	0.13	100	3.20	08
phenylalanine	100	2.70	09	99.1	2.75	0.22	100	2.73	0.17
phosphate	100	3.24	0.64	98.2	3.26	0.63	98.6	3.20	0.54
pimelic acid	100	2.48	0.15	98.2	2.48	0.15	98.6	2.47	0.20
pinitol	55.9	2.11	0.39	53.6	2.09	0.45	49.3	2.10	0.43
proline	52.9	1.99	0.49	55.5	2.18	0.58	64.8	2.22	0.49
pseudo uridine	0	1.48	0.16	0.9	1.49	0.30	4.2	1.56	0.46
psicose	26.5	1.72	0.55	25.5	1.76	0.74	22.5	1.73	0.68
ptolylglucuronide	41.2	1.98	0.42	41.8	1.98	0.39	38.0	1.96	0.52

putrescine	32.4	1.90	0.29	37.3	2.14	0.67	33.8	2.03	0.62
pyrogallol	11.8	1.62	0.30	11.8	1.71	0.31	12.7	1.67	0.29
pyroglutamic acid	97.1	3.55	0.37	99.1	3.75	0.45	100	3.71	0.39
pyrophosphate	100	3.35	0.27	99.1	3.34	0.30	100	3.36	0.28
pyruvic acid	52.9	2.04	0.44	51.8	2.19	0.61	54.9	2.15	0.50
quinic acid	32.4	1.84	0.55	28.2	1.87	0.57	38.0	1.98	0.63
quinolinic acid	0	1.54	0.20	0	1.56	0.20	1.4	1.61	0.26
raffinose	0	1.48	0.23	4.5	1.51	0.32	4.2	1.53	0.24
ribitol	50	2.17	0.66	51.8	2.20	0.67	66.2	2.41	0.65
ribonic acid	0	1.60	0.19	5.5	1.60	0.26	2.8	1.59	0.31
ribose	0	1.45	0.25	1.8	1.46	0.31	5.6	1.46	0.44
saccharic acid	20.6	1.54	0.48	16.4	1.58	0.39	19.7	1.60	0.45
salicylaldehyde	73.5	2.14	0.35	70.9	2.16	0.25	81.7	2.26	0.25
salicylic acid	61.8	2.09	0.23	71.8	2.16	0.37	71.8	2.16	0.37
serine	97.1	2.92	0.64	98.2	3.23	0.79	100	3.19	0.72
serotonin	2.9	1.47	0.24	4.5	1.59	0.47	5.6	1.62	0.47
shikimic acid	97.1	2.27	0.23	98.2	2.40	0.38	97.2	2.37	0.38
sinapinic acid	14.7	1.88	0.18	16.4	1.90	0.11	21.1	1.91	0.14
sophorose	0	1.54	0.26	0	1.47	0.33	1.4	1.38	0.44
sorbitol	100	2.53	0.17	96.4	2.67	0.45	98.6	2.65	0.36
succinic acid	100	2.58	0.16	98.2	2.61	0.27	100	2.64	0.25
sucrose	97.1	2.52	0.37	91.8	2.64	0.59	98.6	2.67	0.49
tagatose	67.6	2.15	0.32	70.9	2.31	0.54	73.2	2.31	0.51
tartaric acid	0	1.40	0.33	0.9	1.50	0.24	2.8	1.54	0.35
threitol	61.8	2.07	0.28	58.2	2.04	0.26	64.8	2.14	0.40
threonic acid	2.9	1.45	0.34	4.5	1.51	0.37	7.0	1.62	0.43
threonine	52.9	2.07	0.35	62.7	2.22	0.50	62.0	2.25	0.45
thymine	0	1.49	0.26	0.9	1.44	0.30	1.4	1.42	0.29
trehalose	97.1	2.42	0.25	98.2	2.46	0.24	94.4	2.41	0.25
triethanolamine	58.8	2.10	0.28	77.3	2.25	0.35	67.6	2.12	0.26
tryptophan	64.7	2.08	0.23	56.4	2.14	0.35	70.4	2.18	0.30
tyrosine	94.1	2.31	0.25	90	2.45	0.47	93.0	2.44	0.41
UDP-glucuronic acid	38.2	1.78	0.41	54.5	1.86	0.46	70.4	2.06	0.42
uracil	14.7	1.74	0.32	14.5	1.72	0.36	26.8	1.81	0.39
urea	97.1	3.12	0.44	99.1	3.35	0.55	100	3.32	0.57
uric acid	0	1.42	0.29	4.5	1.56	0.30	5.6	1.52	0.46
uridine	2.9	1.54	0.25	0	1.52	0.26	0	1.51	0.22
urocanic acid	2.9	1.55	0.28	9.1	1.65	0.41	7.0	1.68	0.25
valine	97.1	2.87	0.31	99.1	3.06	0.47	100	3.08	0.38
vanillic acid	8.8	1.54	0.30	10	1.55	0.39	21.1	1.65	0.34
xanthine	0	1.45	0.19	1.8	1.45	0.28	1.4	1.47	0.29
xanthosine	0	1.42	0.37	3.6	1.51	0.32	4.2	1.50	0.35
xylitol	58.8	2.01	0.12	52.7	2.06	0.26	50.7	2.05	0.27
xylonic acid	0	1.40	0.26	0.9	1.38	0.27	1.4	1.41	0.31
xylonic acidisomer	2.9	1.59	0.18	2.7	1.63	0.26	4.2	1.68	0.26
xylose	100	2.23	0.11	97.3	2.25	0.18	98.6	2.29	0.29
xylulose	0	1.55	0.20	0	1.49	0.19	2.8	1.55	0.32
